# Beyond the screen: Exploring the dynamics of social media influencers, digital food marketing, and gendered influences on adolescent diets

**DOI:** 10.1371/journal.pdig.0000729

**Published:** 2025-02-05

**Authors:** Ashley Amson, Mariangela Bagnato, Lauren Remedios, Meghan Pritchard, Soulene Sabir, Grace Gillis, Elise Pauzé, Christine White, Lana Vanderlee, David Hammond, Monique Potvin Kent

**Affiliations:** 1 Interdisciplinary School of Health Sciences, University of Ottawa, Ottawa, Canada; 2 School of Epidemiology and Public Health, University of Ottawa, Ottawa, Canada; 3 School of Nutrition, Centre NUTRISS (Nutrition, santé, société), Québec, Canada; 4 School of Public Health Sciences, University of Waterloo, Waterloo, Canada; Jordan University of Science and Technology, JORDAN

## Abstract

Adolescent obesity remains a public health concern, exacerbated by unhealthy food marketing, particularly on digital platforms. Social media influencers are increasingly utilized in digital marketing, yet their impact remains understudied. This research explores the frequency of posts containing food products/brands, the most promoted food categories, the healthfulness of featured products, and the types of marketing techniques used by social media influencers popular with male and female adolescents. By analyzing these factors, the study aims to provide a deeper understanding of how social media influencer marketing might contribute to dietary choices and health outcomes among adolescents, from a gender perspective, shedding light on an important yet underexplored aspect of food marketing. A content analysis was conducted on posts made between June 1, 2021, and May 31, 2022, that were posted by the top three social media influencers popular with males and female adolescents (13–17) on Instagram, TikTok, and YouTube (N = 1373). Descriptive statistics were used to calculate frequencies for posts containing food products/brands, promoted food categories, product healthfulness, and marketing techniques. Health Canada’s Nutrient Profile Model was used to classify products as either healthy or less healthy based on their content in sugar, sodium, and saturated fats. Influencers popular with males featured 1 food product/brand for every 2.5 posts, compared to 1 for every 6.1 posts for influencers popular with females. Water (27% of posts) was the primary food category for influencers popular with females, while restaurants (24% of posts) dominated for males. Influencers popular with males more commonly posted less healthy food products (89% vs 54%). Marketing techniques varied: influencers popular with females used songs or music (53% vs 26%), other influencers (26% vs 11%), appeals to fun or coolness (26% vs 13%), viral marketing (29% vs 19%), and appeals to beauty (11% vs 0%) more commonly. Influencers popular with males more commonly used calls-to-action (27% vs 6%) and price promotions (8% vs 1%). Social media influencers play a role in shaping adolescents’ dietary preferences and behaviors. Understanding gender-specific dynamics is essential for developing targeted interventions, policies, and educational initiatives aimed at promoting healthier food choices among adolescents. Policy efforts should focus on regulating unhealthy food marketing, addressing gender-specific targeting, and fostering a healthy social media environment for adolescents to support healthier dietary patterns.

## Author summary

In this study, we explored the role of social media influencers—who have become powerful figures in shaping adolescents’ behaviors—in promoting food products and brands on platforms like Instagram, TikTok, and YouTube. We analyzed the content of posts made by top influencers popular with adolescent boys and girls between June 2021 and May 2022 to see how often food items were featured, what kinds of food were promoted, and the marketing strategies used. Our findings revealed clear gender differences: influencers popular with boys promoted more unhealthy foods and employed distinct marketing techniques, while those popular with girls more often featured healthier options like water. This research sheds light on the types of food content adolescents are exposed to on social media, emphasizing the influence of these digital figures. Our study offers valuable insights for policymakers and educators, providing a foundation to develop gender-sensitive approaches that encourage healthier eating habits among adolescents.

## Introduction

Adolescent obesity remains a public health concern in Canada and abroad [1]. Findings from the 2017/2018 *Health Behaviour in School-aged Children (HBSC) survey* in Europe and Canada found that 21% of adolescents aged 11–15 years were classified as overweight or having obesity based on self-reported height and weight data [2]. This prevalence was higher for boys than girls (25% vs 16%, respectively) [2]. In Canada, the prevalence of obesity among adolescent boys 12–17 years, is higher than for adolescent girls (16.2% versus 9.3%) [3]. Poor dietary patterns are a contributing factor.

Current adolescent dietary intakes consist of high levels of ultra processed foods that are elevated in salt, sugar, and saturated and trans fats [4]. Sex differences related to dietary intake have also been reported. For instance, data from 2015 shows that Canadian males aged 14–18 years consumed a mean intake of 91.7 g of free sugars (e.g., all mono- and di- saccharides added to foods by the manufacturer, plus sugars naturally present in honey, syrups, and fruits juices/juice concentrates) while their female counterparts consumed 68.3 g [5]. Further, males aged 14–18 also consumed more daily sodium than their female counterparts (3320 mg vs 2350 mg) [6]. Results from systematic reviews repeatedly show that exposure to unhealthy food marketing influences adolescent food preferences, short-term intake, and purchase requests, thereby increasing their risk of obesity and other chronic diseases [7].

Adolescents are susceptible to food marketing due to their stage of neuro-cognitive development and their impressionable nature [8]. This makes them an ideal target for food companies looking to foster brand loyalty, which may carry into adulthood and create life-long customers [9]. Food companies have been reported to exploit these vulnerabilities over digital marketing platforms including on social media applications, websites, and online game sites [9–12].

Unhealthy food and beverage (henceforth known as food) marketing is prevalent on digital platforms, creating a high level of exposure for youth [7]. Nearly half of adolescents aged 14–15 years in Canada use their personal digital devices (e.g., smartphones, tablets, laptops, and desktop computers) for leisure purposes, spending 3 or more hours a day on these devices [13]. Online digital media use data from 2019 indicated that 50.2% of girls aged 12–17 years checked social media multiple times daily compared to 41.5% of boys, and 19.7% of girls and 11.8% of boys reported constantly checking their social media apps [14]. Recent data from Canada estimated that adolescents aged 12 to 16 years were exposed to an average of 189 food marketing exposures per week on social media, which translates to over 9000 exposures per year [15]. Food is currently the second most marketed commodity on social media and social media influencers are increasingly becoming an avenue for exposing adolescents to instances of food marketing [16].

Social media influencers (SMIs) are a type of celebrity who acquire online followers by creating or reposting curated social media content [17]. The credibility and trust gained by SMIs have led many companies to add them to their digital marketing strategies [18]. Globally, food marketing promoted on influencer social media pages has been documented. A British study reviewed two YouTube influencer’s popular with youth aged 5–15 and found 3,571 branded food and beverage cues in 380 of their YouTube videos. On average, these youth were exposed to 29.9 food cues per hour [19]. The same study also conducted nutrient profiling, categorizing food and beverage cues into three groups—healthy, less healthy, and miscellaneous. Less healthy foods had the greatest prevalence at 49.4% of the food cues [20,21].

In the ever-evolving landscape of social media, SMIs play a significant role in shaping trends, opinions, and lifestyles, particularly among adolescent audiences [21]. To date, the frequency of food marketing content posted by SMIs popular with adolescents has not been examined in a Canadian context, nor has it been examined from a gender perspective. Examining this content from a gender lens is important as gender, regarded as both a social construct and a determinant of health, has the capacity to foster beneficial or harmful behaviors [22]. Moreover, gender’s influence on social and cultural norms has implications on dietary choices [23] and has shown variations in responses, perceptions, exposure, and content to food marketing amongst boys and girls [24–26]. For example, in a scoping review, six out of nine studies indicated that male and female children and adolescents exhibit distinct responses to specific marketing techniques, including promotions, the use of cartoon characters or sports celebrities in food packaging, and the provision of toys as premiums [26]. While these studies did not specifically address digital media, it can potentially be inferred that boys and girls respond differently to marketing strategies.

Additionally, a recent study explored how adolescents engaged with unhealthy food and beverage marketing in online settings, from a gender perspective [24]. A notable theme was the influence of social media influencers in shaping adolescent dietary preferences [24]. Observed gender differences were noted with boys preferring male influencers, primarily athletes and rappers, while girls followed a mix of both male and female influencers, and a wider breadth of influencer type (e.g., actors, musicians, and pop culture figures). Participants expressed a strong level of trust in the opinions of these influencers and a high likelihood of being swayed by their marketing efforts [24].

Given such findings, the objectives of this study were to determine the frequency of social media posts containing instances of food product/brand content to identify the most promoted food categories, to examine the healthfulness of the featured products, and to identify the marketing techniques being utilized by SMIs popular with male and female adolescents.

## Methodology

This descriptive observational study used a content analysis, based on secondary data from survey results [27], to explore SMI posts containing food products and brands. No human participants were directly involved in this study, so ethical approval was not required.

### Identification of influencers

The top three SMIs popular with male adolescents (13–17 years) (n = 1224) and the top three SMIs popular with female adolescents (13–17 years) (n = 1002) were selected based on data from the 2021 *International Food Policy Study for Youth* [27]. Recruitment for the *International Food Policy Study for Youth (IFPS)* involved participants (N = 2226) living in Canada. Participants were recruited through parents/guardians enrolled in a commercial panel to complete online surveys in English or French. Participants were asked their sex at birth and responded to an open-text question asking, “Who are your three favourite social media stars, TikTokers, or YouTubers?”. Overall, 61% (n = 1364; 743 males and 621 females) named at least one SMI and were included in this analysis. SMIs mentioned in the responses were ranked from most mentioned to least. The following SMIs were most popular with females: 1) Charli D’Amelio (mentioned by 2.9% of female participants), 2) Squeezie (mentioned by 2.1% of female participants), and 3) Mr. Beast (mentioned by 1.9% of female participants). The following SMIs were most popular with males: 1) Mr. Beast (mentioned by 4.6% of male participants), 2) PewDiePie (mentioned by 2.8% of male participants), and 3) Markiplier (mentioned by 1.3% of male participants). Due to overlap of one SMI, posts from a total of five SMIs were included in this study.

### Social media post collection

Publicly available social media posts including pictures and videos, dated between June 1, 2021, and May 31, 2022, from the Instagram, TikTok, and YouTube accounts of the five identified SMIs were analyzed. These social media platforms were chosen as they are the most used by adolescents [28]. Posts were collected by the research team between October 2022 and December 2022. YouTube videos longer than 60 minutes were excluded for feasibility reasons and due to the volume of YouTube videos, only a 50% random sample of YouTube videos were selected using a randomized generator. Instagram “stories” were also excluded from the analysis as they only appear for 24 hours. Overall, 1373 posts (n = 1260 unique, unweighted) were included in this study.

### Content analysis

All SMI posts from the selected year were reviewed by one of five research assistants for instances of food products or brands (e.g., a unique name, logo, or symbol that identifies a specific food product or line of products). Both verbal (e.g., no product or brand actually shown, just mentioned verbally) and visual mentions of food products and brands were identified. Posts containing instances of branded food products or brands were reviewed to identify the presence of marketing techniques. Marketing techniques were identified using a coding manual that was adopted from Mulligan et al. and other relevant literature (S1 Table) [29,30]. Mulligan et al.’s coding manual was developed based on World Health Organization guidelines and primary research that examined child-appealing marketing techniques [29]. The authors identified marketing techniques from 133 publications and organized them into a thematic inventory of 117 distinct techniques [29]. For the current analyses, the coding manual was adapted for adolescents, consistent with its use in other previous research with adolescents [25]. Before coding commenced, a subsample (n = 25) of posts were coded by all five research assistants and compared. The inter-rater reliability was calculated to be 94.5% using Cohen’s Kappa, indicating substantial agreement [31]. Any discrepancies in coding were discussed as a group with a majority ruling.

### Food Categories and Nutrient profiling

Food products were organized into 17 categories, which were adapted based on previous research by Potvin Kent et al. [15]. These categories encompassed a range of items, including bread; sweet baked goods or desserts; candy and chocolate; breakfast cereal; dairy; meat and entrees (e.g., fish, poultry, and meat products); fruit and vegetables; energy drinks; regular soft drinks; diet soft drinks; other sweetened beverages (e.g., sweetened coffee or tea, hot chocolate, fruit drinks); water; snacks; restaurants (e.g., fast food and sit-down); food delivery services; condiments, seasonings, oils and dressings; and other (e.g., gum).

Food products were categorized using Health Canada’s 2018 Nutrient Profile Model (NPM), which is based on the regulatory thresholds for which ‘low in’ claims can be made for sodium, sugars and saturated fat, and has been proposed for future marketing-related regulation [32]. Food products were categorized as healthy (not a concern from an advertising perspective) or less healthy (of concern from an advertising perspective) based on whether they exceeded established thresholds for sugars, sodium, and saturated fats. Nutritional information for each product was collected using the following sources of information in order of priority: the food company’s Canadian website, a Canadian grocery store website (e.g., Loblaws or Walmart), or the company’s American website. A registered dietitian (EP) reviewed the collected nutrition data and assisted with the NPM classification.

### Data analysis

Descriptive statistics, using Microsoft Excel, were used to calculate the following frequencies for each social media platform, stratified by SMI popularity according to the survey participant’s sex: 1) posts containing food products and brands; 2) promoted food category; 3) product healthfulness; and 4) number and type of marketing techniques. Of note, frequencies were weighted for YouTube videos by multiplying each YouTube post by two in order to account for the 50% randomly selected sample of videos. Due to one of the SMIs being popular with both males and females, statistical analyses could not be undertaken, as the samples were not independent. Rates were also calculated by dividing the number of posts by the number of food marketing instances.

Often, sex and gender can be conflated [33]. This paper uses both sex and gender, depending on the content. Participants in the *IFPS* were asked what their sex was at birth, resulting in a male/female comparison. Gender data was not collected in the *IFPS*. Additionally, statistics at the beginning of this paper relating to obesity and food consumption also use data based on sex. Sex, a biological term, refers to physiological features and is often used when discussing health problems [34]. However, the discussion section of this paper will use gender to describe and infer how the content SMIs share on their social media accounts may affect different genders who are exposed to the content. Gender is being used as opposed to sex in this instance as it is a social construct and can affect societal norms and behaviours, such as dietary patterns [22]. The comparison of content posted by social media influencers popular among male and female audiences reveals differences that are rooted in socio-cultural rather than biological factors. Consequently, any observed differences in the types of products advertised or marketing techniques used by influencers favored by boys versus girls may contribute to gender-based disparities in dietary habits and health outcomes.

## Results

### Frequency of food brands/products

As shown in Table 1, SMIs popular with females posted 831 times across the three social media apps examined over the year with 136 posts (16% of total posts) containing a food product/brand. SMIs popular with males posted 542 times with 217 posts (40% of total posts) containing a food product/brand. On average, SMIs popular with males featured 1 food product/brand for every 2.5 posts compared to SMIs popular with females who featured 1 food product/brand for every 6.1 posts. YouTube had the highest rate of featured food products/brands for both males and females (males: 1 food product/brand for every 1.9 posts; females: 1 food product/brand for every 3.4 per posts, respectively), followed by Instagram for males (1 food/product for every 8.8 posts) and TikTok for females (1 food/product for every 6.0 posts).

**Table 1:** Weighted frequencies of collected food product/brand related posts on YouTube, Instagram, and TikTok between June 1, 2021, and May 31, 2022, posted by the top three most popular SMIs with adolescent males and females aged 13–17.

	SMI popular with females			SMI popular with males		
Social media platform	Posts collected n (%)	Food product/brand posts n (%)	Rate of posts per food product	Posts Collected n (%)	Food product/brand posts n (%)	Rate of posts per food product
YouTube^a^	103 (12)	30 (22)	3.4	392 (72)	202 (93)	1.9
Instagram	214 (26)	20 (15)	10.7	53 (10)	6 (3)	8.8
TikTok	514 (62)	86 (63)	6.0	97 (18)	9 (4)	10.7
**Total**	**831 (100)**	**136 (100)**	**6.1**	**542 (100)**	**217 (100)**	**2.5**

Frequencies displayed are weighted.

^a^A random sample of 50% of YouTube posts were analyzed.

SMIs popular with females had 136 posts containing food products/brands with 83% featuring products and 17% featuring exclusively brands, whereas SMIs popular with males had 217 posts containing food products/brands with 97% featuring products and 3% featuring exclusively brands.

### Frequency of products by food category

Among SMIs popular with females, the most featured food categories were water (27%), restaurants (24%), and snacks (18%) (Table 2). For SMIs popular with males, the most featured food categories were restaurants (24%), energy drinks (18%), and snacks (15%). Posts by SMIs popular with males more commonly featured breakfast cereals (5% vs 0%); energy drinks (18% vs 1%); regular soft drinks (5% vs 3%); and other sweetened beverages (17% vs 7%). Whereas posts by SMIs popular with females were more likely to feature water (27% vs 0%). No SMI posts included bread, dairy, or fruits and vegetables. The top three most common brands (i.e., did not include a food product) featured in posts by SMIs popular with females include Starbucks (n = 11, 14%), McDonald’s (n = 5, 7%), and Mr. Beast (n = 3, 4%). The most common products featured in posts include MyMuse Enhanced Water (n = 29, 9%), Dunkin Donuts Iced Coffee (n = 13, 4%), and Coca Cola soft drink original (n = 11, 3%). For SMIs popular with males, the top three most common brands featured in posts included Gfuel (n = 29, 32%), McDonald’s (n = 6, 7%), and Mr. Beast (n = 3, 3%). The most common products featured in posts include Coca Cola soft drink original (n = 7, 6%), Feastables chocolate bars (n = 4, 3%), and G Fuel energy drink powder (n = 3, 2%).

**Table 2:** Weighted frequencies of posts containing food products, by category, on YouTube, Instagram, and TikTok between June 1, 2021, and May 31, 2022, posted by the top three most popular SMIs with adolescent males and females aged 13–17.

	SMI popular with females				SMI popular with males				
Food category	YouTube^a^ n (%)	Instagram n (%)	TikTok n (%)	Total n (%)	YouTube^a^ n (%)	Instagram n (%)	TikTok n (%)	Total n (%)	Total not stratified by sex n (%)
Restaurants (e.g., fast food and sit-down)	8 (36)	6 (40)	15 (18)	**29 (24)**	44 (22)	2 (33)	4 (44)	**50 (24)**	**79 (24)**
Snacks	2 (9)	1 (7)	19 (23)	**22 (18)**	32 (16)	0 (0)	0 (0)	**32 (15)**	**54 (16)**
Other sweetened beverages	2 (9)	2 (13)	4 (5)	**8 (7)**	34 (17)	1 (17)	0 (0)	**35 (17)**	**43 (13)**
Energy drinks	0 (0)	0 (0)	1 (1)	**1 (1)**	38 (19)	0 (0)	0 (0)	**38 (18)**	**39 (12)**
Candy and chocolate	8 (36)	5 (33)	4 (5)	**17 (14)**	12 (6)	2 (33)	2 (22)	**16 (8)**	**33 (10)**
Water	2 (9)	0 (0)	30 (36)	**32 (27)**	0 (0)	0 (0)	0 (0)	**0 (0)**	**32 (10)**
Sweet baked goods/desserts	0 (0)	0 (0)	3 (4)	**3 (3)**	10 (5)	0 (0)	1 (11)	**11 (5)**	**14 (4)**
Regular soft drinks	0 (0)	0 (0)	3 (4)	**3 (3)**	10 (5)	0 (0)	0 (0)	**10 (5)**	**13 (4)**
Breakfast cereal	0 (0)	0 (0)	0 (0)	**0 (0)**	10 (5)	0 (0)	0 (0)	**10 (5)**	**10 (3)**
Condiments, seasonings, oils, and dressings	0 (0)	0 (0)	3 (4)	**3 (3)**	2 (1)	0 (0)	1 (11)	**3 (1)**	**6 (2)**
Diet soft drinks	0 (0)	0 (0)	1 (1)	**1 (1)**	2 (1)	0 (0)	0 (0)	**2 (1)**	**3 (1)**
Meat and entrees (e.g., fish, poultry, and meat products)	0 (0)	0 (0)	0 (0)	**0 (0)**	0 (0)	1 (17)	1 (11)	**2 (1)**	**2 (1)**
Other (e.g., gum)	0 (0)	0 (0)	0 (0)	**0 (0)**	2 (1)	0 (0)	0 (0)	**2 (1)**	**2 (1)**
Food delivery services	0 (0)	1 (7)	0 (0)	**1 (1)**	0 (0)	0 (0)	0 (0)	**0 (0)**	**1 (0.3)**
**Total**	**22 (18)**	**15 (13)**	**83 (69)**	**120 (100)**	**196 (93)**	**6 (3)**	**9 (4)**	**211 (100)**	**331 (100)** *

^a^A random sample of 50% of YouTube posts were analyzed. Frequencies displayed are weighted.

*Percentages add up to over 100% due to rounding.

### Healthfulness

**Fig 1:**
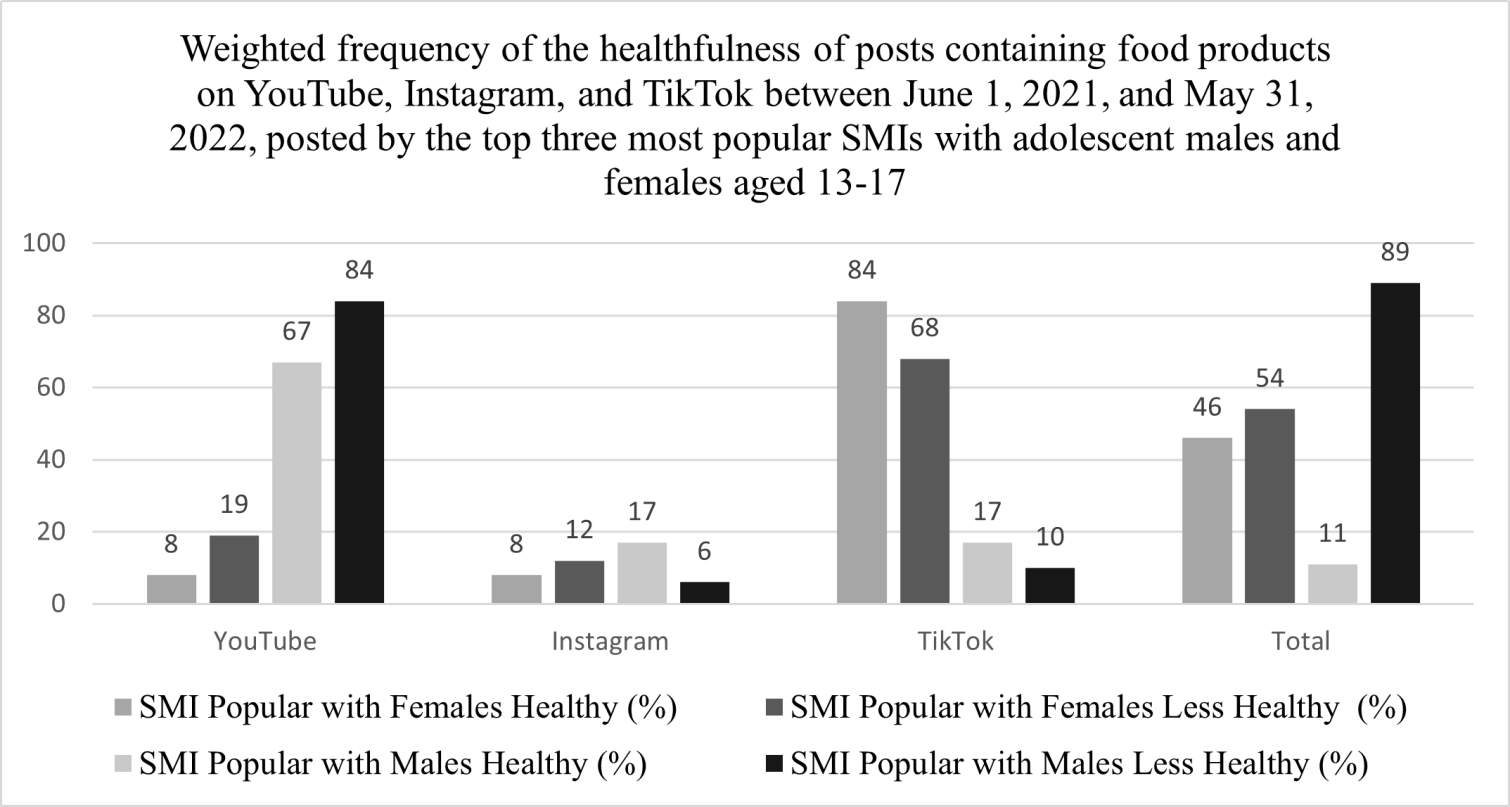
Weighted frequency of the healthfulness of posts containing food products on YouTube, Instagram, and TikTok between June 1, 2021, and May 31, 2022, posted by the top three most popular SMIs with adolescent males and females aged 13–17. ^a^A random sample of 50% of YouTube posts were analyzed. Frequency of products and brands displayed are weighted.

Across all platforms, 54% of food products featured in posts by SMIs popular among females were classified as less healthy compared to 89% for SMIs popular with males (Fig 1). For SMIs popular with females, 68% of less healthy food products were posted on TikTok followed by 19% on YouTube, and 12% on Instagram. Conversely, 84% of posts containing healthy food products were posted on TikTok by SMIs popular with females. Of the less healthy food products posted by SMIs popular with males, 84% were posted on YouTube, 10% on TikTok, and 6% on Instagram.

### Marketing techniques

Overall, the most common marketing techniques for both SMI groups were depictions of the product/brand in a positive or neutral context (female: 97%; male: 83%, respectively) and the product being consumed (female: 59%; male: 47%, respectively) (Table 3). SMIs popular with females more commonly used songs or music (53% vs 26%), the use of other influencers (26% vs 11%), appeals to fun or coolness (26% vs 13%), viral marketing (29% vs 19%), appeals to beauty (11% vs 0%), and the presences of teens (10% vs 4%). Comparatively, SMIs popular with males more commonly used calls-to-action (27% vs 6%) and price promotions (8% vs 1%).

**Table 3:** Weighted frequency of marketing techniques used in all posts featuring food products/brands on YouTube, Instagram, and TikTok between June 1, 2021, and May 31, 2022, posted by the top three most popular SMIs with adolescent males and females aged 13–17.

	SMI popular with females	SMI popular with males
	Total	Total
	*N = 136	*N = 217
	n (%)	n (%)
Unusual product appearance	12 (9)	13 (6)
Unusual product flavour	13 (10)	23 (11)
Positive or neutral context	132 (97)	181 (83)
Product consumed	80 (59)	103 (47)
Sponsorship disclosure	22 (16)	32 (15)
Presence of teens	14 (10)	9 (4)
Adult-child situations	1 (1)	0 (0)
Child or teen language	0 (0)	2 (1)
Child themes	2 (1)	2 (1)
Licensed characters	1 (1)	1 (0.5)
Other cartoon characters	1 (1)	0 (0)
Use of actors	0 (0)	2 (1)
Use of other influencers	36 (26)	23 (11)
Appeals to fun or coolness	35 (26)	29 (13)
Appeals to healthfulness	5 (4)	9 (4)
Appeals to athleticism	1 (1)	0 (0)
Appeals to beauty	15 (11)	0 (0)
Appeals to energy	1 (1)	2 (1)
Appeals to achievement	3 (2)	0 (0)
Songs or music	72 (53)	57 (26)
Appealing graphic effects	18 (13)	31 (14)
Animations	1 (1)	3 (1)
Price promotions	1 (1)	18 (8)
Calls-to-action	8 (6)	59 (27)
Incentives/giveaways	5 (4)	3 (1)
Limited time/seasonal item	0 (0)	1 (0.5)
Viral marketing	39 (29)	42 (19)

*% are a proportion of total posts containing food products/brands (N).

There were no instances of the following marketing techniques being used: presence of children; adult-teen situations; teen themes, spokes characters; use of athletes; use of musicians; appeals to social enhancement; appeals to sex; cross-promotions; games or activities; corporate social responsibility; and advercation (e.g., ad is linked to online educational content) (not presented in the table).

## Discussion

This study provides preliminary insights into the frequency of posts featuring food products/brands, the most featured food categories, the healthfulness of products, and the marketing techniques most often used by SMIs popular with adolescents, while considering gender. The results highlight differences in posts among SMIs popular with males and females, including the frequency of posts containing food products/brands and the variance of marketing techniques, as well as the proportion of energy drinks and unhealthy food products being shared by SMIs popular with males.

### Frequency, food categories, and healthfulness

Adolescents are heavily exposed to food marketing on various mediums [35]. SMIs are burgeoning in popularity among adolescents and have an influence on adolescents’ dietary patterns [19]. In this study, SMIs popular with males posted one food product/brand for every 2.5 posts, whereas those popular with females posted one food product/brand for every 6 posts. Further, most food products promoted by both SMI groups were unhealthy. Differences amongst SMI groups were noted, with SMI popular with females featuring 54% of food products as less healthy or “of concern from an advertising perspective”, compared to 89% for SMI popular with males. These results hold significance, considering research highlights that boys have an increased susceptibility to the influence of food marketing compared to girls [26]. The notable prevalence of unhealthy food content and more frequent instances of food marketing by social media influencers popular among males may indicate a troubling trend in heightened exposure to unhealthy food products for boys, potentially leading to adverse short- and long-term health consequences [7].

We also observed that SMIs popular with a certain sex posted their content to platforms predominantly used by individuals of the same sex/gender. For instance, SMIs popular with males predominantly created content on YouTube while SMIs popular with females predominantly created content on TikTok, aligning with trends that a higher proportion of boys use YouTube compared to girls with the opposite trend being true for TikTok [36]. Trends and patterns have been observed for platform preferences between boys and girls [37]. Boys are more drawn to YouTube content, particularly gaming videos, sports highlights, or technology-related videos. TikTok focuses on visual and short-form content, which appeals to girls, as they tend to enjoy sharing photos, videos, fashion, beauty, and creative content [38]. A SMIs choice in platform selection could reflect an awareness of demographic preferences, suggesting they are intentional in reaching certain audiences. Food companies may also capitalize on this awareness, selecting specific SMIs who are on certain platforms to promote their products. The relationship between the popularity of a SMI, their platform choice, and the demographic composition of platform use emphasizes the importance of considering platform-specific dynamics in influencer marketing strategies.

In the contemporary landscape of social media, the promotion of food products by SMIs can shape consumer perceptions, preferences, and behaviors. For both SMI groups, restaurants, including fast food, were among the top three most featured food products. It is not surprising that restaurants, including fast-food, were common among both SMI groups. These findings align with other research that highlights the frequency of fast-food marketing in digital and traditional media [19,35,39].

For SMIs popular with males, the most featured products were energy drinks, restaurants, and snacks. While this study did not investigate actual exposure, this suggests that boys may be exposed to energy drink marketing more frequently than girls. Other research has noted how male adolescents reported greater exposure to online energy drink marketing compared to their female counterparts [40]. Energy drink marketing is widespread, particularly on social media and livestreaming platforms like Twitch, Facebook gaming, and YouTube, platforms that are more favored by boys than girls [40]. Energy drink marketing is presently directed primarily towards boys, and this approach appears to be effective, as reflected by higher levels of energy drink consumption among boys compared to girls [40–42].

### Marketing techniques

A variety of marketing techniques were used by each SMI group. The most common across all platforms and amongst both groups were displaying the product using a positive or neutral context and the product being consumed. These techniques are not surprising, given the nature and intent of marketing. The ubiquity of food products/brands presented in a positive or neutral context found in our results aligns with other literature [19,43]. These results are noteworthy, as positive presentations of unhealthy food by SMI can translate into a positive view of these foods and brands by adolescents, leading to unhealthy dietary patterns [18].

There were differences between the techniques more commonly featured by SMIs popular with males versus females. The use of songs or music, the use of other influencers, appealing to fun or cool, viral marketing, appeals to beauty, and the presence of teens were marketing techniques more commonly used by SMIs popular with females, whereas SMIs popular with males more commonly featured calls-to-action (prompts for additional actions beyond the initial advertisement [44], and price promotions. Creating techniques that appeal to an intended audience can include the use of gender stereotypes and norms [25]. Recent research has found that a small sample (n = 139) of boys and girls aged 12 to 16 were exposed to statistically different marketing techniques based on their gender, suggesting targeted marketing [25]. The public health impact of targeted marketing is significant, as it can create health disparities [45].

Traditional gender roles and societal expectations can shape preferences and interests, resulting in SMIs using these as marketing techniques. It is no surprise that appeals to beauty were only present among SMIs popular with females and not males. Influencers will tailor their content to align with the interests of their target audiences and adapt their techniques to the platform preferences of those audiences [46]. For example, the use of music and beauty appeals is more commonly found on TikTok, a platform more popular with girls, while calls to action are emphasized more on YouTube, which is favoured by boys [36]. Understanding the content and marketing messages endorsed by SMIs can provide insights into the interplay between influencers and audiences, supporting the discovery of more gendered nuances of social marketing.

### Power of SMIs

The influence of SMIs on consumer behavior has become a subject of growing interest. SMIs represent a unique and dynamic marketing technique in themselves, serving as relatable and influential figures who engage with audiences on a personal level [47]. SMIs perceived authenticity and direct connection with followers distinguish them from traditional advertising methods. Social media platforms have provided influencers with unprecedented reach and access to diverse audiences, enabling them to impact consumer choices and preferences [20].

SMIs often receive monetary compensation from food companies as part of a companies’ marketing campaign. For example, one of the SMI’s examined within this study, Charli D’Amelio, is sponsored by Dunkin’ Donuts [48]. Food companies frequently collaborate with influencers to leverage their large followings and high engagement rates among young audiences [49]. This collaboration typically involves sponsored posts, product placements, or promotional giveaways, where influencers are paid to showcase or endorse food products on platforms like Instagram, TikTok, or YouTube [19]. Companies capitalize on the influencers’ ability to shape trends and create authentic connections with their audience, making it an effective way for food companies to target adolescents. The use of SMIs by companies to advertise their food products illustrates how businesses recognize the effectiveness of SMIs in reaching and engaging specific audience segments, further highlighting their powerful role in shaping trends and consumer habits. These collaborations may be contributing to unhealthy eating habits, increasing the risk of obesity among adolescents who are particularly vulnerable to the persuasive power of social media and peer influence [47].

A recent qualitative study that explored how adolescents engage with unhealthy food marketing in online settings found the most frequently mentioned marketing technique was the use of a SMI [24]. Nearly all participants in the study reported following at least one influencer. Participants also expressed a strong inclination to purchase products endorsed by a SMI due to the trust they placed in them, which by extension, translated to a positive reaction to the promoted products [24]. This observed trust aligns with several findings underscoring the significant influence SMIs wield over adolescents’ food preferences [47]. This degree of trust is what links adolescent food choices, increased recall, and consumption of unhealthy foods, to SMI food promotion [21]. Furthermore, adolescents showed a preference for food posts when they belonged to a celebrity, influencer, or peer, opposed to posts from a food company [21]. Given this, the food industry could be strategically leveraging SMIs as a marketing tool to promote their products, revealing a calculated approach in targeting specific populations, such as adolescents.

Gender roles and societal expectations can influence food choices [50]. For instance, if male SMIs popular with males promote indulgence in unhealthy and high-calorie foods, their male followers may be more inclined to adopt these eating habits to conform to that SMI. Similarly, female influencers who endorse specific food choices have the potential to persuade females, potentially shaping the consumption behaviors of their female followers. An interesting observation from a small sample study revealed that female audiences engaged with both female and male influencers, while males predominantly followed only male influencers [24]. If this observed trend is generalizable, it suggests that females might be exposed to a more diverse range of marketing techniques and products compared to their male counterparts. This nuanced relationship between gender, SMIs, and food choices is a complex interaction of relatability, targeted marketing, and social norms. Recognizing these factors can help adolescents become more mindful media users, critically evaluating the content they encounter on social media platforms.

### Implications

Targeted food marketing raises ethical concerns, particularly regarding children’s rights under the UN Convention on the Rights of the Child (CRC) [51]. According to the CRC, children are defined as individuals up to the age of 18 [52]. Marketing nutritionally poor foods infringes on their right to health, adequate nutrition, and privacy [52]. Article 24 of the CRC mandates countries to combat malnutrition and protect children’s health by limiting unhealthy food marketing [52]. Digital marketing also exploits children’s personal data, violating their privacy (Article 16) [52]. Personalized targeting makes food marketing content highly immersive and specific [16]. Data, such as an individual’s gender, can be used to curate content by reinforcing gender stereotypes—targeting boys and girls differently, which in turn can amplify health and social inequalities. Article 36 of the CRC calls for the protection of children from all forms of exploitation, including manipulative marketing practices [52]. SMIs often blur the lines between entertainment and advertising, making it difficult for children to recognize promotional content [49]. This is problematic, as SMI often promote unhealthy foods, leading to poor dietary habits, increasing the risk of obesity, diabetes, and other health conditions [21]. A more ethical approach would involve more stringent government-led policies to restrict children’s exposure to unhealthy food marketing.

The landscape of food marketing has transformed, with SMIs becoming influential intermediaries between brands and consumers [47,53]. This shift is particularly significant when targeting adolescents, a demographic highly susceptible to marketing influences who are actively engaged in social media [8]. In safeguarding adolescents against the multifaceted influences of social media, particularly exposures to SMIs, a three-pronged strategy may include parental involvement, media literacy, and policy.

Parental involvement can be a protective factor for obesity prevention whereby they endorse and model healthy eating patterns, foster critical thinking about food marketing, and engage in open and informed discussions about food [54]. Research initiatives from the USA and Jamaica emphasize the role of parent–child communication as a catalyst for obesity prevention [55]. This was further reinforced by a small qualitative study that revealed participant’s belief in the protective influence of parents on adolescents’ food choices [24]. This notion was mostly cited by girls in the study [24].

Parental influence on attitudes toward food and healthy eating exhibit gender-based differences [54]. Parents typically show greater concern for the diets and body weight of their daughters compared to their sons [56]. Healthy eating is not as emphasized or discussed by men and consequently, they may be less aware of their dietary patterns and its consequences [56]. Understanding how gender shapes adolescents’ responses to food marketing, how SMIs may be targeting their child, and recognizing current food norms can guide parents in providing tailored support to their child’s unique needs, fostering better evaluative skills in response to food marketing.

The second facet of this strategy involves bolstering media literacy, where the combination of family discussions, critical analysis of media messages, and educational components about media use collectively empower adolescents to assess and discern information [55]. Educating adolescents about social media, particularly SMI marketing, is needed to enhance their ability to recognize these persuasive tactics [57]. While media literacy programs have demonstrated some effectiveness, several challenges exist, such as the absence of a standardized marketing literacy curriculum, difficulties integrating interventions into the school setting due to existing curricular constraints, and teachers requiring specific knowledge and skills to implement such a curriculum, necessitating their own refinement of marketing literacy [57]. Nevertheless, more robust media literacy among adolescents can cultivate awareness and critical thinking about their social media exposures, with particular attention to gender nuances and the targeted efforts of food companies and SMIs based on gender stereotypes and norms.

Lastly, policies designed to monitor and restrict unhealthy marketing practices that effect adolescents is needed, as currently children are the focal point when it comes to marketing restriction policies [11]. Impactful policies need to consider restricting the persuasive power of food marketing, such as influencer and celebrity endorsements. Suggestions have been made to enforce sponsorship disclosures, infrequently seen in our study, to support adolescents in recognizing sponsored content as advertising. A recent study demonstrated how textual sponsorship disclosures on TikTok influencer food marketing helped adolescents recognize the commercial and persuasive intent of the videos [58]. However, while disclosure enhanced awareness, it did not lead to more critical attitudes or altered product choices, likely due to adolescents limited executive functioning and susceptibility to social influences [58]. Another important consideration is advocating for increased accountability from social media platforms in their marketing practices.

The regulation of advertising products such as tobacco and alcohol on social media platforms is predominantly self-regulated, placing the responsibility for content accuracy and legal compliance on businesses [59]. While many platforms enforce age restrictions and other policies for alcohol and tobacco advertising, similar measures for unhealthy food marketing remain scarce [59]. Notably, adolescents continue to encounter alcohol advertisements on social media despite the existence of these policies, raising concerns about their effectiveness [24].

An analysis of 12 popular social media platforms identified only two—Snapchat and YouTube Kids—that have implemented limited restrictions on food advertising [59]. YouTube Kids prohibits all advertisements for food and beverages, irrespective of nutritional quality [59]. However, a 2019 study revealed that the platform still hosted videos featuring food advertisements, the majority of which promoted unhealthy options high in sugar, fat, and salt [60]. Snapchat, on the other hand, has introduced industry-specific guidelines for sectors such as pharmaceuticals, healthcare, diet, and fitness [59]. These guidelines mandate that food advertisements provide accurate descriptions of their characteristics, including health and nutritional claims, but enforcement remains inadequate [59].

The widespread implementation of policies restricting advertising for tobacco and alcohol demonstrates the feasibility of extending such restrictions to unhealthy food marketing. Companies like Disney have already adopted guidelines banning advertisements that promote unhealthy lifestyles, showcasing the potential for platform-driven initiatives to safeguard younger audiences from harmful marketing practices [61].

Moreover, developing comprehensive food marketing policies necessitates an understanding of gender dynamics. Gender analyses of food marketing content is important for evaluating gender-specific messaging and biases, guiding policies that discourage the perpetuation of gender portrayals in marketing, and for uncovering targeting patterns so that policies can be responsive to the emerging marketing trends impacting different gender groups. Recognizing adolescents’ heightened susceptibility to marketing, regulations addressing gender dynamics in marketing strategies targeted at this demographic, including content restrictions based on nutrient profiling models, are essential [62]. By integrating these policy considerations, an environment can be created that actively addresses the complex intersections between gender, dietary choices, and marketing influences. This approach reflects a commitment to fostering equity and promoting health-conscious decision-making in the realm of food marketing. Combing parental involvement, media literacy, and policy can contribute to synergistically creating a healthy social media environment for adolescents, mitigating the potential negative impacts of SMI exposures.

### Strengths and Limitations

To our knowledge, this is the first study to examine social media posts across three platforms shared by SMIs popular with Canadian adolescents. It is important to highlight that while our study focused on SMIs popular with adolescents in Canada, many of these influencers are popular in other countries. This suggests that the potential impact and reach of marketing by these SMIs extends beyond national borders.

This study is also subject to several limitations, including that actual exposure was not captured. Instead, this study captured the frequency or potential exposure to food marketing by examining posts of SMIs popular with male and female adolescents. It was also not feasible to code every YouTube video, and as such, a 50% sample was used and weighted frequencies were applied. This in turn only provides an estimate for the total number of posts containing food products and brands on YouTube and may not be reflective of the actual total. This limitation may affect the results by introducing potential biases, as the sample may not be fully representative of the broader social media content landscape. Consequently, certain marketing techniques or trends could be underrepresented or overrepresented, leading to skewed findings and limiting the generalizability of the results. Similarly, the results could be shaped by platform-specific biases; for instance, YouTube’s higher usage among males and TikTok’s greater appeal to females might impact the marketing strategies and content that are examined. Additionally, our study may not be representative of all potential posts as the researchers reviewed posts dated from June 1, 2021, to May 31, 2022, but they were collected from October to December 2022. This allowed the potential for SMIs to delete posts that the researchers could not account for. Further, due to the popularity of one of the SMIs being prevalent with both males and females, statistical analyses could not be undertaken, as the samples were not independent. This limits our ability to determine whether differences in marketing strategies used by influencers popular with males and females are statistically significant. The SMIs included in this study also represent a small sample size with a disproportionate SMI sex representation (4 male SMIs and 1 female SMI), which could impact the generalizability of findings particularly the limited female representation that may not fully capture the diversity of female influencer content. Additionally, the presence of Mr. Beast in both samples could skew the comparisons. Lastly, posts did not contain information on whether SMIs were compensated for their promotion of said food products or brands. Without knowing if influencers received payment or incentives, it becomes difficult to assess the potential bias in their endorsements. Nevertheless, this study offers insights into what adolescents may be exposed to while viewing posts by their preferred SMI. By understanding how and how often influencers promote unhealthy food products/brands, we can gain a deeper understanding of the evolving social and cultural landscapes shaped by digital media. Future research could investigate how SMI content impacts the eating and purchasing behaviors of adolescent boys and girls.

## Conclusion

Our research sheds light on the food marketing content posted by SMIs popular among adolescent boys and girls. The differences in SMI preferred platforms, food categories, and marketing techniques suggest SMI are tailoring their content to specific demographics. This adaptation implies an awareness of audience preferences based on sex/gender, influencing both content creation and promotional strategies. The disparities in food posts among influencers emphasizes the importance of recognizing gender-specific dynamics in influencer marketing. Understanding how influencers engage with their audiences, considering gender nuances can guide more effective and responsible marketing practices to protect adolescents. Further studies exploring the implications of these differences on consumer behaviors, health outcomes, and societal perceptions will be instrumental in advancing our understanding of the multifaceted influence of SMIs in shaping food marketing trends.

## Supporting information

S1 Table Marketing technique descriptions and examples. (DOCX)

S1 Data SMI final master database. (XLSX)

S2 Data Top 10 influencers data. (XLSX)
