# Supplementary material for: Beyond the screen: Exploring the dynamics of social media influencers, digital food marketing, and gendered influences on adolescent diets
Source: PLOS Digit Health. 2025 Feb 5;4(2):e0000729. doi: 10.1371/journal.pdig.0000729 (PMC11798478; doi:10.1371/journal.pdig.0000729)
Supplement: S1 Table — (DOCX) [file pdig.0000729.s001.docx]

**S1 Table.** Marketing technique descriptions and examples

| **Name of Technique** | **Description and example** |
| --- | --- |
| Unusual product appearance | Ad depicts a product’s shape or colour that is unconventional or unusual for that specific product. E.g., If goldfish crackers (typically fish shaped and yellow in color) come out with a rainbow appearance. |
| Unusual product flavour | Ad promotes a product flavour that is unconventional or unusual for that specific product, or a flavour that is not a ‘real’ or ‘discernable’ flavour. E.g., Coke coming out with cherry flavoured coke. This could also include the presentation of the flavour in a ‘negative’ way that may appeal to youth – tastes crazy, weird, sour, whacky. |
| Context | What is the overall context of the post?  E.g., The user describes the product in a positive way or seems positive with the product (smiling) or the user is not a fan or the product or displays a negative tone about the product (frowns/scowls). |
| Product consumed | Does the influencer consume the product/brand (e.g., drink or eat) |
| Sponsorship disclosure | The influencer displays or mentions a sponsorship disclosure. E.g., This product is brought to you by “McDonald’s/X” or I’ve teamed up with “Tim Hortons to bring you …”. If not said verbally, usually there is a hashtag tied to the sponsorship or something may be written in the description.  This needs to be explicit. Even if the influencer is obviously in a partnership with the brand (E.g., Justin Bieber and Time Hortons), if they do not say that the ad is sponsored then it is not considered sponsorship disclosure. |
| Presence of children | Child is present in the ad.  A child is anyone under the age of 12. |
| Presence of teen | Teen is present in the ad |
| Adult-child situation | Post features situations that play on the parent-child relationship or other authority-based relationship (e.g., coach-child, teacher-child). |
| Adult-teen situation | Post features situations that play on the parent-teen relationship or other authority-based relationship (e.g., coach-teen, teacher-teen). |
| Child or teen language | Post uses language that is associated with children, that is frequently used by children, or that is directed at children.  E.g., “OMG” “dope” etc. |
| Child themes | Post uses themes, designs, colours, images, or other elements of audiovisual design that are commonly associated with children such as fantasy, magic, mystery, suspense, adventure, zoo animals, virtual worlds, etc. This could include references to, or the incorporation of, popular trends in children’s interests of preferences, which may vary year-to-year or based on geographic location. This needs to be a deliberate inclusion of child themes to market the product. |
| Teenager themes | Post uses themes, designs, colours, images, or other elements of audiovisual design that are commonly associated with teens such as themes linked to high school, social media, ‘hanging-out’, popularity, fashion, risk-taking, independence, etc. This could include references to, or the incorporation of, popular trends in teens interests or preferences, which may vary year-to-year or based on geographic location. This needs to be a deliberate inclusion of teen themes to market the product. |
| Spokes characters | A fictional/cartoon character that is defined by a set of human attributes and characteristics to give the brand a unique personality. E.g., Tony the Tiger, Pillsbury Doughboy, etc. |
| Licensed characters | A license character involves licensing the rights from the owner of the cartoon character to place images on a product. E.g., Using Spiderman on a luncheables package. |
| Other cartoon characters that are not spokes or licensed | Post features generic cartoon characters, cartoon children/teens, animals, or imaginary/virtual creatures etc. that are not branded or licensed characters, celebrities, or cross-promotions to other media. |
| Use of Athletes | An athlete(s) is present or referenced in the post. E.g., Lebron James, Hailey Wickenheiser, etc. |
| Use of Actors | An actor(s) is present or referenced in the post. E.g., Chris Hemsworth, Nathalie Portman, etc. |
| Use of Musicians | A musician(s) is present or referenced in the post. E.g., Ed Sheeran, Ariana Grande, etc. |
| Use of other Influencers | Another influencer is present or referenced in the post. E.g., Charli D’Amelio featuring Addison Rae, Mr. Beast featuring Jake Paul, etc. |
| Appeals to fun or cool | Post makes appeals to the food or beverage item being fun or funny, having fun while eating the product, being happy, humour or coolness/novelty. This could include depictions of the food itself doing something fun, or depictions of the food in motion, for example, a cookie diving into milk, or candies ‘exploding’ out of ice cream, juggling food products, someone spinning Oreos on their finger, etc. It can also be part of the name such as “Fun Dip” or “Kool Kreatures”. |
| Appeals to social enhancement | The post highlights the product’s ability to enhance making friends, peer acceptance, or being social with others. E.g., Coca-Cola advertisements that feature people giving friends Coke’s with their names on them. |
| Appeals to athleticism | The post highlights the products attributes to athleticism, referring to its ability to boost one’s strength, speed, or sports performance or features individual(s) doing athletic activities with the product.  E.g., People snowboarding while drinking Mountain Dew. |
| Appeals to sex | The post includes aspects of romance, sex, or sexuality to market a product. E.g., Paris Hilton wearing a bikini while eating at a Carl’s Jr Burger. |
| Appeals to beauty | The post includes aspects of beauty or attractiveness to market the product. E.g., An influencer doing their make-up while consuming a drink or food product. |
| Appeals to healthfulness | Post makes explicit appeals related to the healthfulness or nutritional quality of the product, its ability to promote wellness, growth, strength, or physical activity. The post can also make implicit appeals to health or nutrition, such as the product being displayed alongside “healthy foods” (e.g., fresh fruit being depicted in an ad for breakfast cereal), or the product being shown consumed by children while participating in physical activity. E.g., the post includes health and nutrition claims/symbols, as well as claims or symbols referring to the product being organic or natural. |
| Appeals to energy | The influencer references or utilizes energy as a selling point e.g., “Red bull gives you wings” |
| Appeals to achievement or success | The post highlights the product’s ability to help with achievement or accomplishment or the influencer indicates this product has helped them become successful. E.g., Mento’s that show problem solving or achievement because of consuming the product. Grey Poupon showing status or achievement. |
| Appealing graphic effects | Visual effects are used in the post or to market the product. E.g., explosions, lights, fast cutting, slow motion, dynamic images, etc. Graphic imagery is used in the post or to enhance the display of the product. E.g., bright colours, eye-catching backgrounds, fonts, etc.  I.e., eye-catching elements |
| Songs or music | Music is used in the post. E.g., songs, jingles, sound effects, etc. |
| Animations | Animations are used in the post to make the product appealing. E.g., the use of cartons to interact with the product. |
| Cross promotions | Post features cross-promotions to movies/sporting event/TV show etc. other than one of the types of characters or celebrities described above. E.g., the ad features aspects of a well-known fictional world, without specifically including the fictional characters.  Note: these may appear in addition to the presence of any characters described above.  Does NOT include promotion of other food or non food brands (e.g., Uber Eats) or to other food products. |
| Price promotions | Post includes a price-promotion or premium, including discounted prices on other merchandise included with the purchase of a food or beverage product. E.g., Charlie D’Amelio offers a discount on make-up if you buy Takis. |
| Incentives and giveaways | Post promotes contests, prizes, or giveaways available with or without purchase. E.g., Addison Rae will give away $1000 to the first 100 people that comment on her post. |
| Calls to action | Post encourages consumers to either participate in a campaign, visit a product/brand/company website, social media, or games-based brand website or promote opportunities to “join”, “become a member”, complete a quiz, poll, or survey. Encourage the user to spend more time with the brand. E.g., Charli D’Amelio will encourage you to check out her linked tree and sign up for an account.  I.e., sending the user/viewer to an additional website or link |
| Corporate responsibility | Post makes appeals to sustainability, philanthropy or contributing to other social causes. E.g., For every like on this post I (the influencer) will donate a dollar to cleaning up beaches. |
| Viral marketing | Prompts viewers to engage with the brand by commenting, replying, sharing information with their peers (peer-to-peer marketing), re-posting content to their own feeds, tagging friends, or using specific hashtags. E.g., “Be sure to share this post with all of your friends”.  A hashtag of a company is displayed either in the description, comments section, or on the screen.  E.g., #wendys #invisalign.  Includes tagging brands or other influencers (>10k followers).  Emphasis is on sharing with others. |
| Games | Presence of games or activities within the post (including on packaging, marketing display, etc.). E.g., Mr. Beast is playing a video game while drinking Coke. Also includes tie-ins with games (e.g., UNO deal with Pizza Pizza) |
| Advercation | Ad is linked to online educational content. E.g., a chocolate ad where the influencer teaches you about where the cocoa beans came from and how they were harvested etc. |
| Limited time/seasonal item | Is product promoted as a limited time or seasonal item (e.g., “Starbucks Peppermint Mocha only here for the holiday season!”) |
